# Finished Genome Sequence of the Laboratory Strain *Escherichia coli* K-12 RV308 (ATCC 31608)

**DOI:** 10.1128/genomeA.00971-14

**Published:** 2014-11-20

**Authors:** Peter M. Krempl, Juergen Mairhofer, Gerald Striedner, Gerhard G. Thallinger

**Affiliations:** a Bioinformatics Group, Institute for Knowledge Discovery, Graz University of Technology, Graz, Austria; b Department of Biotechnology, BOKU University of Natural Resources and Life Sciences, Vienna, Austria; c Austrian Centre of Industrial Biotechnology (ACIB GmbH), Graz, Austria

## Abstract

*Escherichia coli* strain K-12 substrain RV308 is an engineered descendant of the K-12 wild-type strain. Like its ancestor, it is an important organism in biotechnological research and is heavily used for the expression of single-chain variable fragments. Here, we report the complete genome sequence of *E. coli* K-12 RV308 (ATCC 31608).

## GENOME ANNOUNCEMENT

The prototrophic *Escherichia coli* K-12 RV308 strain was derived from the RV strain by P1 transduction in the Ptashne lab (1). The original *E. coli* K-12 RV strain was engineered by P. Roy Vagelos in 1962 during his stay at the Institut Pasteur, Paris, France, by mating the *E. coli* K-12 3000X74V2 Hfr strain with the *E. coli* K-12 C600 strain (M. Malamy, personal communication). According to Maurer et al. (1), *E. coli* K-12 RV308 carries the following modifications: *su*^−^, Δ*lacX74*, *gal IS II*::OP*308*, and *strA*. However, the actual genome sequence remains to be elucidated.

The genome of *E. coli* strain K-12 substrain RV308 was sequenced using a combination of next-generation sequencing methods. A first draft assembly based on the sequences of an 8-kbp paired-end library (Roche 454 GS FLX Titanium, 779,412 reads, for a total of 123.1 Mb; 26-fold coverage; ZMF, Medical University of Graz, Austria) generated with Newbler 2.6 consisted of 126 contigs, 71 of which were joined into a single circular scaffold. The gaps resulting from repetitive sequences were resolved by *in silico* gap filling. The remaining gaps were closed by PCR, followed by Sanger sequencing, yielding a draft genome of 4,583,642 bp. To improve the quality of the sequence by eliminating the 454 sequencing errors in homopolymer stretches, the genome was subsequently sequenced using the Illumina paired-end method (Illumina HiSeq 2000; 16,875,234 reads, for a total of 1.69 Gb; 368-fold coverage; Ambry Genetics, Aliso Viejo, CA). The Illumina reads were aligned to the draft genome with CLC Genomics Workbench 4.7.1 (CLC bio, Aarhus, Denmark). Conflicts in the consensus sequence were resolved by voting.

The genomic structure of RV308 was compared with that of reference *E. coli* K-12 strains, especially MG1655, using Mauve (2), showing that RV308 does not contain genomic regions without a homolog in MG1655. Functional annotation was performed by matching the genes of MG1655 based on sequence similarity, using EcoCyc (3) as a primary data source. Additionally, the origin of replication was predicted with OriginX (4). Clustered regularly interspaced short palindromic repeats (CRISPRs) were predicted with CRT (5).

The final sequence includes 4,585,620 bases, with a G+C content of 50.79%. The genome consists of 4,463 putative genes, 4,245 of which are protein coding. There are seven instances of the ribosomal 5S-23S-16S cluster, an additional 5S rRNA genes, 86 predicted tRNAs, one transfer-messenger RNA (tmRNA), and 91 RNA genes of regulatory or miscellaneous function. Two CRISPRs were detected with 12 and 6 spacers, respectively.

The closest sequenced K-12 strain is *E. coli* MG1655 (6). In relation to MG1655 (GenBank accession no. U00096.3), the RV308 genome shows 8 deletions (65 to 36,218 bp) and 6 insertions (181 to 1336 bp). Specifically, the e14 and CPZ-55 prophages are missing, and the *yah*, *prp*, *cod*, *cyn*, *lac*, and *mhp* operons and one IS*1* element are deleted. The insertions comprise 5 additional IS elements (3 IS*1*, 1 IS*2*, and 1 IS*3*).

### Nucleotide sequence accession number.

The genome sequence for *E. coli* strain K-12 substrain RV308 has been deposited at the European Nucleotide Archive under the accession no. LM995446.
